# Does signal reduction imply predictive coding in models of spoken word recognition?

**DOI:** 10.3758/s13423-021-01924-x

**Published:** 2021-04-14

**Authors:** Sahil Luthra, Monica Y. C. Li, Heejo You, Christian Brodbeck, James S. Magnuson

**Affiliations:** 1 Department of Psychological Sciences, University of Connecticut, Storrs, CT 06269-1020 USA; 2 Connecticut Institute for the Brain and Cognitive Sciences, Storrs, CT USA; 3 BCBL, Basque Center on Cognition Brain and Language, Donostia-San Sebastián, Spain; 4 Ikerbasque, Basque Foundation for Science, Bilbao, Spain

**Keywords:** prediction, spoken word recognition, computational models, cognitive neuroscience

## Abstract

Pervasive behavioral and neural evidence for *predictive processing* has led to claims that language processing depends upon *predictive coding.* Formally, predictive coding is a computational mechanism where only deviations from top-down expectations are passed between levels of representation. In many cognitive neuroscience studies, a reduction of signal for expected inputs is taken as being diagnostic of predictive coding. In the present work, we show that despite not explicitly implementing prediction, the TRACE model of speech perception exhibits this putative hallmark of predictive coding, with reductions in total lexical activation, total lexical feedback, and total phoneme activation when the input conforms to expectations. These findings may indicate that interactive activation is functionally equivalent or approximant to predictive coding or that caution is warranted in interpreting neural signal reduction as diagnostic of predictive coding.

**Supplementary Information:**

The online version contains supplementary material available at 10.3758/s13423-021-01924-x.

## Introduction

Theories of spoken language processing posit that listeners continually engage in predictive processing. Behavioral studies indicate that listeners leverage linguistic information (e.g., lexical, syntactic, semantic) to anticipate upcoming phonemes and words (Allopenna et al., 1998; Altmann & Kamide, 1999; Grosjean, 1980; Kukona et al., 2011; Magnuson et al., 2008; Strand et al., 2018). Likewise, electrophysiological data support the idea of prediction (see Kuperberg & Jaeger, 2016, for review); listeners show differential neural responses to predicted words compared to unexpected words (e.g., Kutas & Hillyard, 1980, 1984), with research suggesting that these responses specifically index a process of pre-activation (DeLong et al., 2005, DeLong et al., 2017; but see Nieuwland et al., 2017, for results suggesting these effects may be weaker than found in prior work).

One mechanism through which predictive processing might be achieved is *predictive coding*. While the term *predictive processing* is often used synonymously with *predictive coding*, the latter has a precise formal definition: Predictive coding is a computational framework wherein the information at a lower level of a model is compared to a generative prediction derived from a higher level (Rao & Ballard, 1999). In Rao and Ballard’s model, this is achieved through two sub-populations of units: neurons that encode the actual input state and error-detecting neurons that compute the deviation between the input and what was expected. Critically, only this prediction error is passed along to other layers for further processing, enabling the model to code incoming information more efficiently. Thus, a property of predictive coding is a reduction in the signal being sent from one layer to another when information is expected (i.e., when there is low prediction error) compared to when information is unexpected.

In functional neuroimaging studies of spoken word recognition, the finding of reduced activation for expected inputs is often taken as diagnostic for predictive coding (e.g., Gagnepain et al., 2012; but see Aitchison & Lengyel, 2017). For now, we take this assumption at face value. If it is the case that a reduction of signal for expected inputs is diagnostic of predictive coding, then observing this pattern in a computational model of spoken word recognition would imply that the model is implementing predictive coding. Note that in these functional neuroimaging studies, predictive coding does not involve a general reduction of activation across all of cortex; rather, if this pattern is observed in *any* part of a network, it is taken as evidence for predictive coding. In the same spirit, we would consider a computational model of spoken word recognition to be consistent with predictive coding if we observe a reduction of signal for expected input *anywhere* in the model.

Intuitively, some computational models may be more likely than others to exhibit this putative hallmark of predictive coding. Simple Recurrent Networks (SRNs), for instance, predict a model’s upcoming states based on its previous states (Elman, 1990), so they necessarily involve predictive *processing*. Furthermore, SRNs involve explicitly computing prediction error during learning, with this error being used to update the model’s weights. Thus, an SRN built to predict upcoming phonemes would arguably also show evidence of predictive *coding*, as described in the Online Supplementary Materials (OSM). By contrast, the TRACE model of speech perception (McClelland & Elman, 1986) is a less likely candidate to show evidence of predictive coding, as it does not involve explicit prediction. Predictive coding models generally involve some form of inhibition to cancel out predicted inputs and propagate prediction errors. However, TRACE incorporates mechanisms that should strengthen predictable inputs: Excitatory feedback connections from higher layers enhance signals consistent with higher level representations (which could, e.g., activate lexically consistent phonemes in advance of direct bottom-up support), and lateral inhibition within layers further enhances dominant signals (what Blank & Davis, 2016, term “signal sharpening”).

The goal of this investigation is to consider whether a reduction of signal for expected inputs should constitute evidence for predictive coding. Based on the logic discussed above, if TRACE exhibits signal reduction for predictable inputs, then this could mean two things – either that the property is not a good diagnostic for predictive coding or that TRACE employs predictive coding in an unanticipated manner. For our simulations, we specifically consider the case of novel word learning, which serves as a useful domain for assessing predictive processing because a listener’s predictions about upcoming phonemes will change as novel words are added to the lexicon. We are guided by a study from Gagnepain et al. (2012), who observed a reduction in the degree of activity in the superior temporal gyrus (STG) when upcoming phonemic segments were less predictable.

Below, we provide a brief overview of the Gagnepain et al. (2012) study in order to define clear empirical targets for subsequent simulations. We then show that, surprisingly, these patterns are observed in the dynamics of the TRACE model. We then discuss the implications of our results and return to the question of whether a reduction in activation for expected inputs is truly diagnostic of predictive coding.

## Empirical target: Gagnepain et al. (2012)

The study from Gagnepain et al. (2012) was largely influenced by work from Gaskell and Dumay (2003), who exposed listeners to novel words (e.g., *cathedruke*) that overlapped with existing source words (e.g., *cathedral*) at onset. Gaskell and Dumay noted that once a novel word like *cathedruke* has been lexicalized, the associated source word (*cathedral*) would be associated with increased lexical competition, as measured by performance in a pause-detection task (since listeners are slower to detect short pauses in spoken words that are associated with many lexical competitors; Mattys & Clark, 2002). When tested on the same day as when they learned the novel word, listeners were able to explicitly indicate what the novel word had been, but their performance on the pause-detection task was not affected, suggesting the novel word had not yet been lexicalized. When tested several days later, however, listeners were slower to detect pauses in source words that had become associated with a novel word, suggesting that the novel words had been lexicalized. Subsequent work in this domain has suggested that sleep-mediated consolidation plays an important role in lexicalizing novel words (Davis & Gaskell, 2009; Dumay & Gaskell, 2007; Palma & Titone, 2020), and a functional magnetic resonance imaging (fMRI) study (Davis et al., 2009) established an association between lexicalization and activation of the STG, with robust activation for items not integrated in the lexicon (unfamiliar nonwords as well as novel words learned shortly before the fMRI scan, which therefore had not been consolidated) and minimal activation for items that had been integrated into the lexicon (source words as well as novel words learned the day before, which therefore had an opportunity to be consolidated).

Building on these previous studies, Gagnepain et al. (2012) suggested that the lexicality effect in the STG might specifically reflect sensitivity to phoneme-level prediction error. In their study, listeners were familiarized with novel words (e.g., *formubo*) that overlapped in onset with source words (e.g., *formula*). The following day, listeners were exposed to additional novel words (e.g., *mushrood*, which overlaps with the existing *mushroom*). An hour later, listeners participated in an MEG session where they performed a pause-detection task on *source *words(e.g., *formula, mushroom*), the trained *novel *words (e.g., *formubo*, *mushrood*), and untrained *baseline *nonwords (e.g., *formuty*, *mushrook*). Note that novel words learned on the first day (e.g., *formubo*) might have benefitted from sleep-mediated consolidation, but novel words presented on the second day (e.g., *mushrood*) could not have.

To quantify prediction error, the authors calculated the frequency-weighted probability of each phoneme given the preceding input. For example, given the input *for…* (/for/), the prediction for the fourth phoneme position would reflect the frequency of all possible continuations in the lexicon (e.g., *forbid*, *forceps*, *foreign*, *formula*). With additional input, fewer phonemes would be possible; for instance, given the input *formu…* (/formju/, the only possible continuation for the seventh phoneme is /l/. Gagnepain et al. assessed probabilities with respect to the *deviation point* (DP), or the point in the stimulus after which the item can be uniquely identified; for *formula*/*formubo*/*formuty*, the DP would be after *formu*-. The authors found that:

1. After the DP, unconsolidated novel words (*mushrood*) and untrained baseline nonwords (*mushrook*) were associated with relatively high prediction error (as calculated in their mathematical model) and relatively high STG signal (as measured with MEG). By contrast, source words (*mushroom*) were associated with low error and low STG signal.
2. After the DP, consolidated novel words (*formubo*) patterned with source words (*formula*); both were associated with low prediction error and low STG signal. However, similar baseline nonwords that had not been presented in training (*formuty*) were associated with high prediction error and STG signal.
3. The influence of consolidated novel words was also seen prior to the DP. If the pre-DP segment matched both a source word and a consolidated novel word (as in the pre-DP segment *formu-*, which matches both *formula* and *formubo*), there was relatively low prediction error (since two lexical entries supported the prediction) and correspondingly low STG activity. By contrast, if the pre-DP segment matched a source word and an unconsolidated novel word (as in the pre-DP segment *mushroo-*, since *mushrood* had not yet been consolidated into the lexicon), there was a higher prediction error (since only one lexical entry supported the prediction) and correspondingly higher STG activity.

In all of these results, as the degree of prediction error varied, so too did the degree of STG activation. Thus, the authors argued that these findings constituted evidence for predictive coding.

### Methods

We re-implemented the mathematical model used by Gagnepain et al. (2012) using a set of 37.6 k words from the English Lexicon Project (Balota et al., 2007) that were ≤ 12 phonemes long. In an initial set of *pre-training* simulations, we computed by-position phoneme probabilities for 54 triples taken from Gagnepain et al. (e.g., source: *formula*, novel: *formubo*, baseline: *formuty*). Note that while we use *formubo* as our example novel word and *formuty* as our example baseline nonword, we in fact ran two simulations and counterbalanced which specific nonword (e.g., *formubo* or *formuty*) served as the novel item and which as the baseline item, with results representing an average across the two simulations. Stimuli had a mean length of 6.3 phonemes, with all source words having six phonemes, and the DP occurred 0–3 positions prior to stimulus offset. To simulate word learning, we simply added a set of novel words to the lexicon and then re-calculated by-position phoneme probabilities. Following the approach of Gagnepain et al., novel words were assigned the same frequency as their associated source word. We also calculated by-position prediction error for each phoneme. Given a prediction of 1.0 for the phoneme /l/ at position 7, prediction error would be 0 if /l/ is indeed encountered. Given any other phoneme at this position, prediction error would be 2.0, since prediction error is summed over all phonemes. For instance, if the predicted probabilities for /l/ and /b/ were 1.0 and 0.0, respectively, but the input was 0.0 for /l/ and 1.0 for /b/, summed absolute error would be 2.0.

### Results

**Fig. 1:**
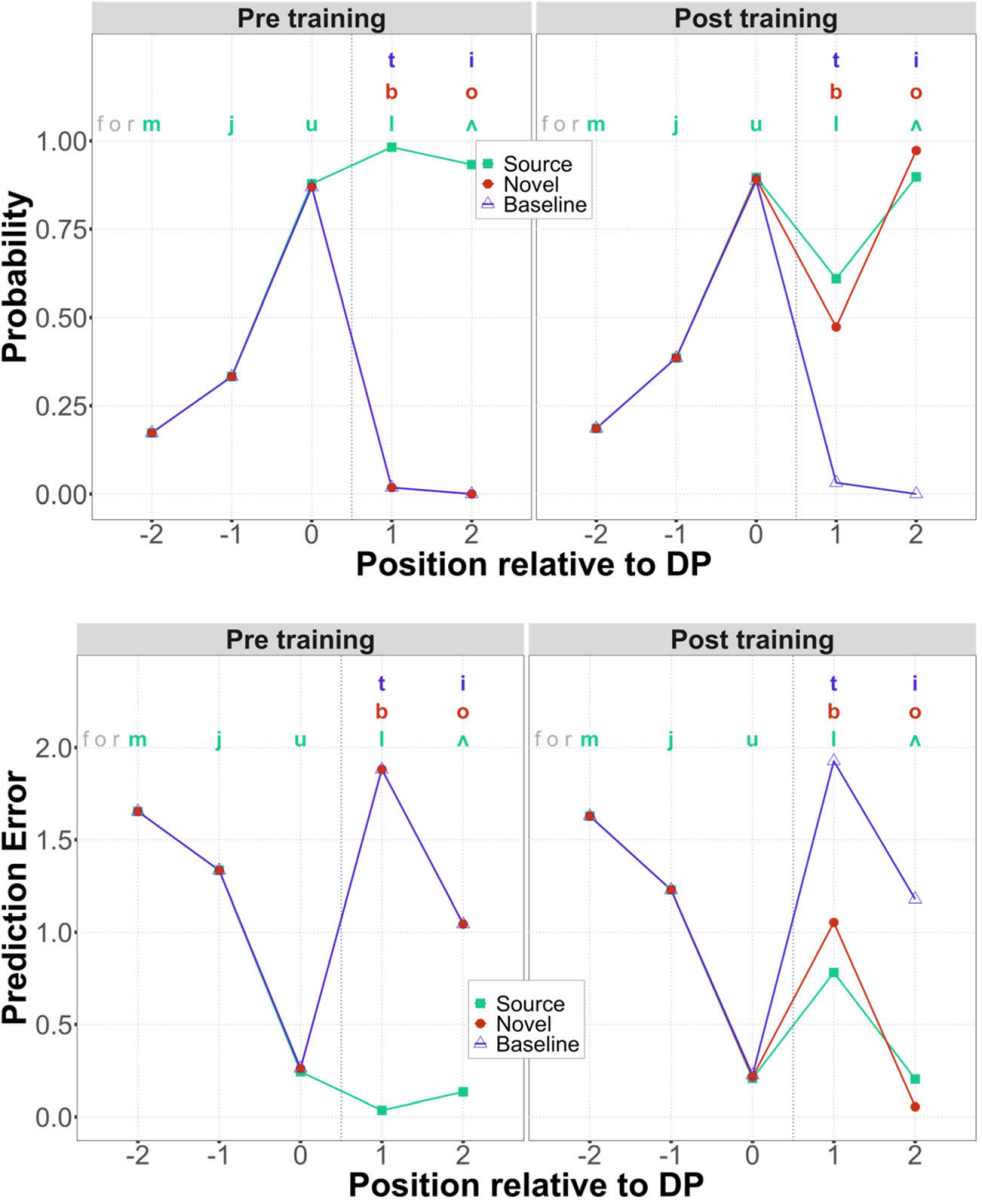
Predicted phoneme-by-phoneme probabilities (top) and derived errors (bottom), pre- (left) and post- (right) training for the mathematical model used by Gagnepain et al. (2012). The x-axis shows position relative to the deviation point, allowing us to align results for all items. Dashed lines between positions 0 and 1 indicate the deviation point. These results constitute empirical targets for subsequent simulations

By-position phoneme probabilities and prediction error are shown in Fig. 1. Because training involved simply adding words to the lexicon, training is analogous to (sleep-mediated) lexicalization in human subjects. Thus, novel words in the *pre-training* simulations are comparable to the unconsolidated novel words in the Gagnepain et al. (2012) study, and novel words in the *post-training* simulations are comparable to the consolidated novel words. Crucially, simulations with our implemented model capture the three key findings from Gagnepain et al. described above:

1. In pre-training simulations, the prediction error for post-DP phonemes is low for source words (-*la* in *formula*) but high for (unconsolidated) novel words (*-bo* in *formubo*) and baseline nonwords (-*ty* in *formuty*).
2. In post-training simulations, the prediction error for post-DP phonemes is low for source words (-*la* in *formula*) and for (consolidated) novel words (-*bo* in *formubo*), but prediction error remains high for baseline nonwords (-*ty* in *formuty*).
3. Novel word learning also has a measurable influence on pre-DP phonemes, as the error for pre-DP phonemes (*formu*-) is slightly higher prior to training (mean error: 1.66) than after training (mean error: 1.62).

These three findings serve as empirical targets for subsequent simulations.

## TRACE simulations

TRACE (McClelland & Elman, 1986) is an interactive activation model with feedforward connections (from features to phonemes to words) as well as excitatory feedback from its word layer to its phoneme layer. This feedback allows for enhanced activation of phonemes that are consistent with lexical knowledge. Because of this “signal sharpening,” TRACE has been characterized as contrastive with models that emphasize prediction error (Blank & Davis, 2016). However, this may be an oversimplification of the model’s dynamics, as TRACE also includes lateral inhibition within each layer. Furthermore, feedback in interactive activation provides a generative model through which predictive processing can occur (Magnuson et al., 2018; McClelland, 2013; McClelland et al., 2014); as TRACE receives input consistent with a particular lexical candidate, the model sends feedback from nodes in the word layer to their constituent phonemes in the phoneme layer, including those phonemes in the word that have not yet been encountered.

Here, we conducted a set of simulations based on the approach of Gagnepain et al. (2012) to test whether TRACE exhibits a reduction of signal when inputs are consistent with expectations.

### Methods

Because TRACE does not include all the phonemes used in the Gagnepain et al. (2012) stimuli, we selected a set of 15 six-phoneme words on which to base our item sets. For each source item (e.g., *partly*; /partli/), we created two related nonwords (/partk^/ and /partsa/) by changing the final two phonemes. In pre-training simulations, we used the 212-word TRACE lexicon. For post-training simulations, we added 15 novel words to the lexicon. While we use /partk^/ as our example novel word and /partsa/ as our example baseline nonword, we in fact counterbalanced which specific nonword was used added to the lexicon during training, and results represent an average of the two counterbalancing sets. For all simulations, we tracked activations of phonemes and words over time as well as the total amount of activation flow between and within each layer. TRACE simulations were conducted using an implementation of TRACE in C (available at https://github.com/maglab-uconn/predictive_coding).

### Results and discussion

**Fig. 2:**
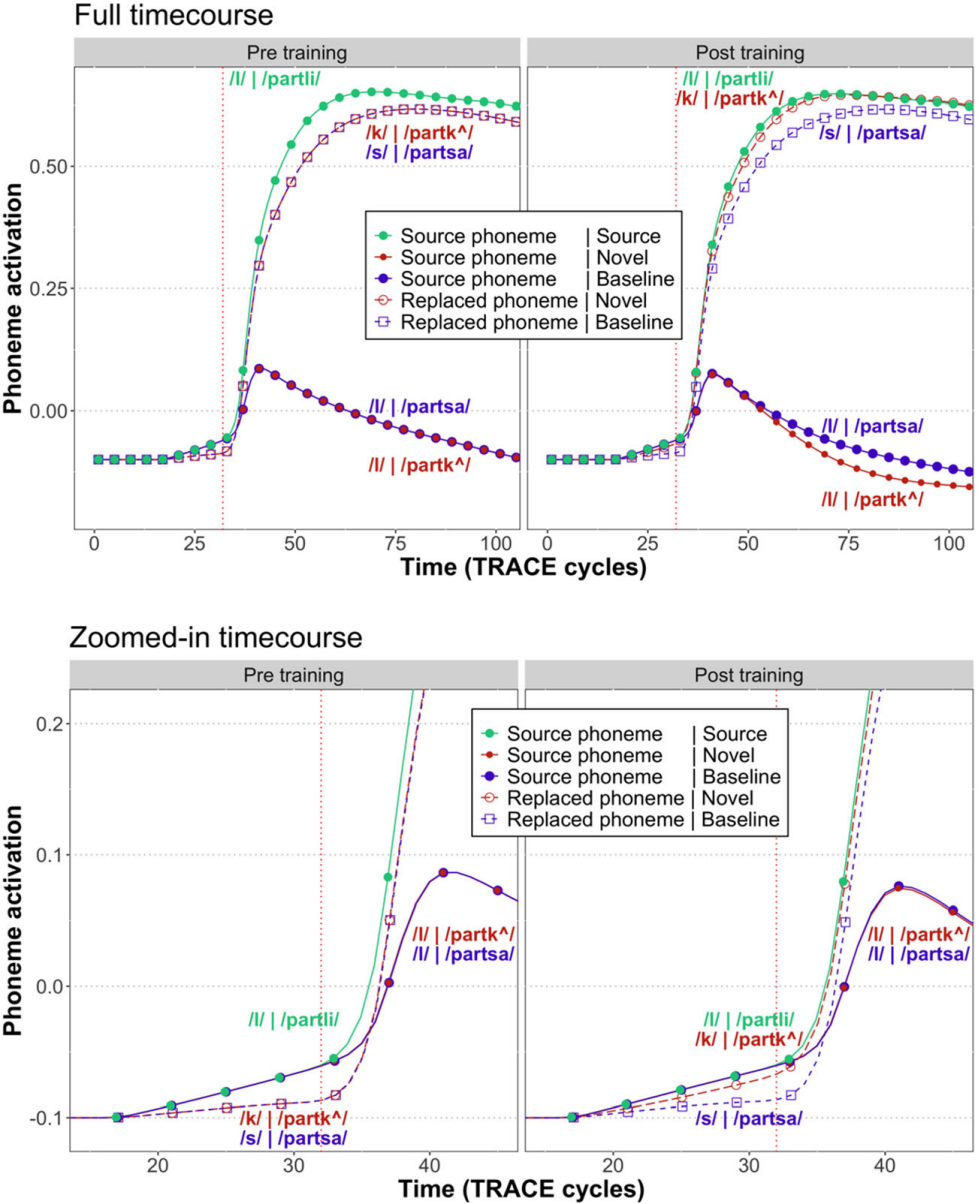
Activation of the first post-deviation point (DP) phoneme (e.g., /l/ in /partli/) in TRACE. The top panel shows the entire time course, whereas the bottom panel shows a zoomed-in view of the cycles immediately prior to the deviation point (indicated by the vertical red dotted line). As shown in the bottom panels, training leads to an increase in the activation of the replaced phoneme in novel words (e.g., /k/ in /partk^/) even before the deviation point, which demonstrates predictive processing in TRACE

Figure 2 shows the activation of the first phoneme after the DP (e.g., /l/ in /partli/). Prior to training, the source phoneme (/l/) achieves the highest activation, owing both to the bottom-up input and to the top-down support from the lexicon. Replacement phonemes (e.g., /k/ in /partk^/, /s/ in /partsa/) are only supported by bottom-up input, leading to a slight disadvantage in the total amount of activation for these phonemes. Adding the novel word (/partk^/) to the lexicon leads to the source phoneme (/l/) and the trained phoneme (/k/) both reaching a comparable degree of activation, as both receive top-down support from the lexical layer as well as bottom-up support from the input.^1^

Evidence for predictive processing is apparent in the bottom panels of Fig. 2, which show a zoomed-in version of the time steps immediately adjacent to the DP. Pre-training, the activation of the predicted phoneme (/l/, solid lines) is higher than the activation of the unexpected phonemes (/k/ and /s/, dashed lines) for approximately 15 cycles prior to the DP. Post-training, there is an increase in the amount of activation for the replacement phoneme in the novel word (/k/, red dashed line) in the cycles prior to the DP. Thus, at both stages of the TRACE simulations, we see activation of anticipated phonemes prior to them being presented in the bottom-up input – clear evidence of predictive processing.

To test for evidence of predictive coding, we tracked the degree of activation at each level as well as the amount of activation flowing between levels. Several indices met some or all of the empirical targets defined above.

**Fig. 3:**
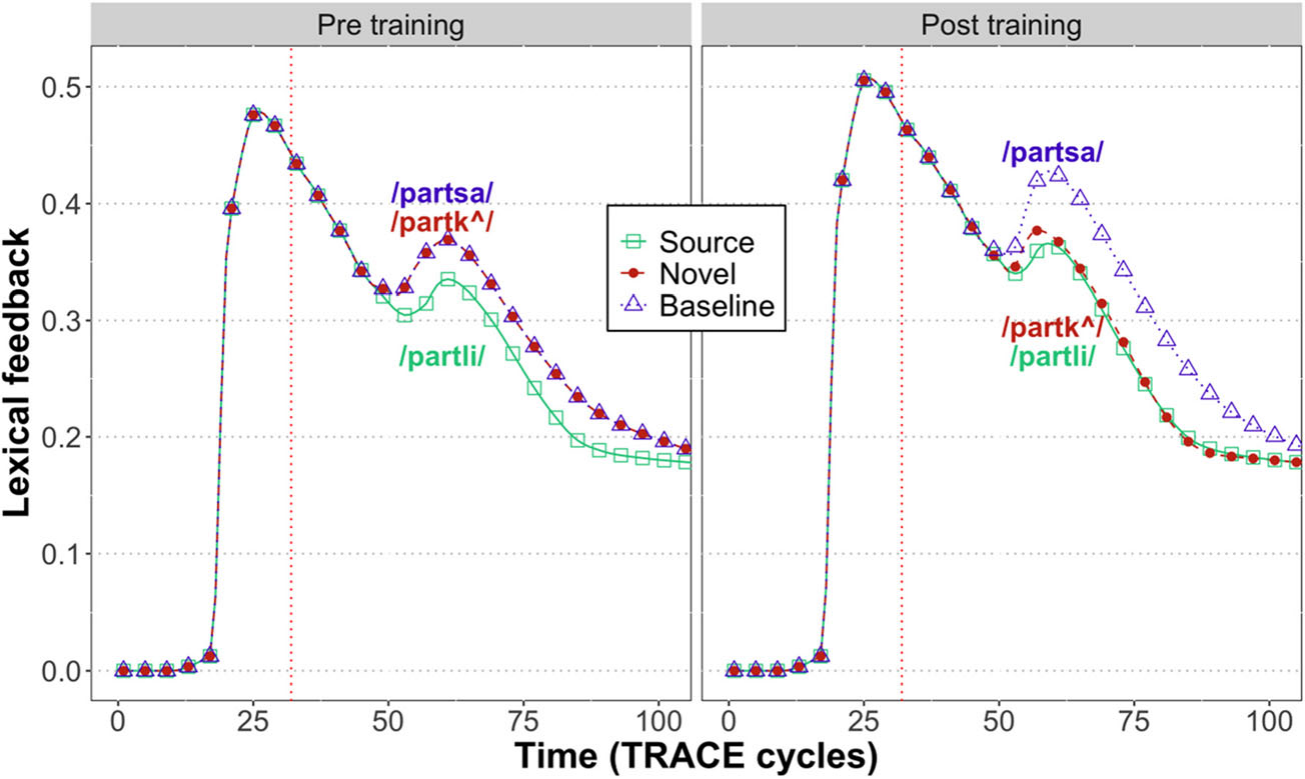
Total lexical feedback over time in TRACE, showing what has been claimed to be a hallmark of predictive coding – robust signal reduction when expectations are met

Figure 3 shows the total amount of feedback from the word layer to the phoneme layer. We observed a reduction of signal for expected inputs insofar as:

1. Prior to training, there was less feedback for source items (e.g., /partli/) compared to both types of nonwords.
2. After novel words (e.g., /partk^/) were added to the lexicon, there was a reduction of total feedback for source words (/partli/) and novel words (/partk^/) relative to baseline nonwords (/partsa/).

However, prior to the DP (red vertical line in Fig. 3), there was an *increase* in the total magnitude of the feedback signal after training (mean: 0.20) compared to beforehand (mean: 0.18). This is inconsistent with a pure prediction error signal, which should be reduced slightly prior to the DP, since the likelihood of the pre-DP sequence, shared by the source word and the novel trained word, has increased. However, during this period, lexical competition is also increased, due to the competition from the added novel word. While Gagnepain et al. (2012) did not see any brain activity tracking lexical competition (quantified through lexical entropy), a subsequent MEG study using continuous speech stimuli found such signals alongside effects of how unexpected a particular phoneme was (phoneme surprisal; Brodbeck et al., 2018). Our findings thus show that the amount of lexical feedback in TRACE prior to the DP may be influenced by lexical competition, though future work is needed to more clearly relate TRACE activity to phoneme surprisal and to cohort entropy.

**Fig. 4:**
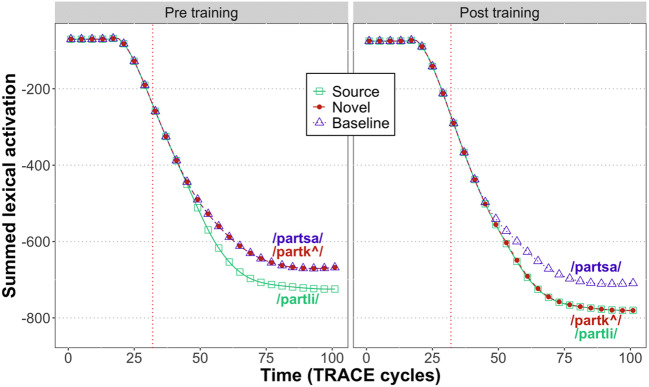
Total amount of activation at the lexical level in TRACE. Unexpected inputs are associated with greater activation than expected inputs.

**Fig. 5:**
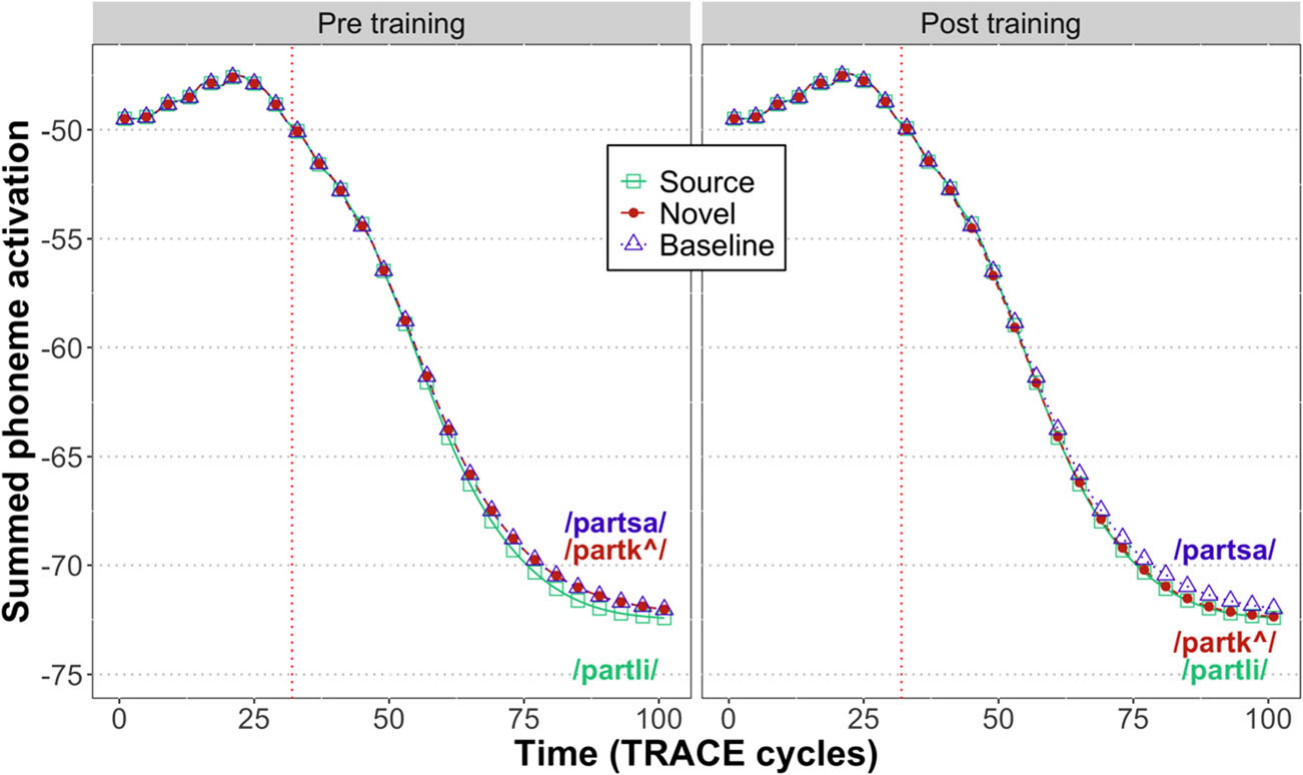
Total amount of activation at the phoneme level in TRACE. Unexpected inputs are associated with greater activation than expected inputs

Figures 4 and 5 show the total activation (summed over all candidates) in the lexical and phoneme layers, respectively. At both levels, we observe reduced activation for expected inputs:

1. Prior to training, there is greater activation for both types of nonwords (/partk^/ and /partsa/) relative to the source word.
2. Following training, there is greater activation for the untrained baseline nonword (/partsa/) relative to both the trained novel word (/partk^/) and the source word (/partli/).
3. In the lexical layer, the degree of pre-DP activation is higher prior to training (mean: -97.3) compared to after (mean: -106.2), consistent with the results of Gagnepain et al. (2012). At the phoneme level, however, the degree of pre-DP activation prior to training (mean: -48.5) is unchanged by training (mean: -48.5).

Our results can be understood by considering that when the bottom-up input is inconsistent with the model’s predictions, there is an increase in the number of activated lexical candidates. For instance, when the unexpected /k/ in /partk^/ is encountered, word units aligned with that phoneme (e.g., a word unit for *carpet* /karp^t/ aligned at position 5 of /partk^/) are also activated.^2^ Even though these candidates are only weakly activated, the increase in the number of supported lexical candidates leads to an increase in the total amount of feedback from the word layer to the phoneme layer and an increase in the total activation of the phoneme layer. In this way, TRACE shows a reduction of signal when it receives input consistent with prior expectations, both in the magnitude of feedback from the lexical layer to the phoneme layer, and in the activations of the phonemic representations themselves.

These findings are also consistent with a previous MEG/EEG study (Gow et al., 2008). In that study, the presentation of a phonetically ambiguous stimulus (a *s/sh* blend heard in a lexically disambiguating context, like *_andal* or *_ampoo*) was associated with increased activity in the STG during a time window associated with lexical processing. Critically, this increase in STG activity was predicted by previous activity in an area associated with wordform processing, the supramarginal gyrus; specifically, there was a Granger-causal relationship between the activity of the supramarginal gyrus and the STG, which was presumed to reflect lexical feedback. Thus, in both the previous work by Gow and in the current set of simulations, the presentation of an unexpected stimulus was associated with an increase in lexical feedback during a relatively early time window and an increase in phoneme-level activation during a later time window.

## General discussion

In previous studies, signal reduction for expected inputs has been viewed as diagnostic of predictive coding. Here, we conducted a set of simulations based on the approach of Gagnepain et al. (2012) and found that TRACE exhibited this sign of predictive coding in the total amount of activation at the lexical level, in the total degree of lexical feedback to the phoneme layer, and in the total amount of activation at the phoneme level. These effects were primarily seen for phonemes following the DP of the stimuli. These findings are striking given that TRACE is not an explicit prediction model and indeed has been described as standing in contrast to models that compute prediction error (Blank & Davis, 2016) due to the “signal sharpening” impact of lexical feedback.

We see at least two ways to interpret our results. One possibility is that the interactive activation framework implemented in TRACE is functionally equivalent (or at least approximant) to a generative Bayesian model (as suggested by McClelland, 2013, McClelland et al., 2014, and Magnuson et al., 2018) and perhaps even to predictive coding. Testing this will require the development of models of spoken word recognition that formally implement predictive coding. Such models must work on real speech (or at least abstract phonetic inputs that are presented over time, as in TRACE), must be validated with a large set of words, and must account for a wide range of behavioral phenomena. While this is a tall order, some progress has been made in this regard, with a growing number of models working on real speech (Kell et al., 2018; Magnuson et al., 2020; Yildiz et al., 2013).

Alternatively, the reduction of signal for expected inputs may not actually be diagnostic of predictive coding, as previously suggested, for instance, by Aitchison and Lengyel (2017). For instance, a reduction of signal for expected inputs would also emerge if neural activity indexed the amount of attention directed toward a stimulus, as unexpected stimuli would elicit more attention. We argue that in the absence of formal models of predictive coding, we must be cautious in interpreting a reduction of signal for expected inputs as diagnostic of predictive coding, whether in neurobiological studies or in computational models. Instead, we suggest that a better diagnostic might be found in considering how the *information content* at different levels of processing changes depending on whether inputs are expected. Two recent neuroimaging studies are particularly inspiring in this regard. In an fMRI study Blank and Davis (2016), the authors examined how much information about the phonological similarity between stimuli was encoded in the activation patterns of superior temporal cortex. More recently, Sohoglu and Davis (2020) examined how well information about the spectrotemporal modulations in the speech signal was represented in the MEG signal. In both studies, the authors observed that the information content of the neural signal tracked the degree of calculated prediction error, a finding that is readily explained by predictive coding frameworks. We believe that the strategy of examining information content will be particularly beneficial for evaluating whether computational models of spoken word recognition are consistent with predictive coding and see this as an exciting direction for future research.

## Supplementary Information

ESM 1 (DOCX 636 kb)

## Footnotes

1 We suspect that the slight reduction in activation of the trained phoneme for novel words (/k/ in /partk^/; red dashed line in Fig. 2) compared to the activation of the source phoneme in source words (/l/ in /partli/; green line) is due to the specific phonological neighborhoods in our stimuli. (Because there are fewer items in the TRACE simulations than in the SRN simulations, idiosyncratic differences due to specific stimuli are likely to be relatively pronounced.) Notably, this slight difference in activation was not observed in a separate set of simulations where we fully counterbalanced the conditions (source, novel, baseline) to which our items (/partli/, /partk^/, /partsa/) were assigned; note that this is in contrast to the present set of simulations, where we only counterbalanced whether /partk^/ or /partsa/ served as the novel word or baseline nonword. We opted to use this simpler counterbalancing scheme to match the counterbalancing approach of the other simulations in this project.

2 The increase in lexical competition also manifested as a delay in activation of the source word (/partli/) after a novel word (/partk^/) was added to the lexicon (Fig. S3, OSM), consistent with the findings of Gaskell and Dumay (2003).
