# Supplementary material for: Does signal reduction imply predictive coding in models of spoken word recognition?
Source: Psychon Bull Rev. 2021 Apr 14;28(4):1381–9. doi: 10.3758/s13423-021-01924-x (PMC8367925; doi:10.3758/s13423-021-01924-x)
Supplement: Supplementary file 1 — (DOCX 636 kb) [file 13423_2021_1924_MOESM1_ESM.docx]

**Supplementary Materials**

***Simple Recurrent Network (SRN) simulations***

Simple Recurrent Networks (Elman, 1990) involve explicit computation of prediction error, which is then used to update model weights during training. We would therefore expect an SRN to show the putative hallmark of predictive coding: a reduction of signal for expected inputs (at least in the signal used to guide learning). Here, we describe a set of simulations with an SRN that provide a useful benchmark against which to contrast the TRACE simulations reported in the main text.


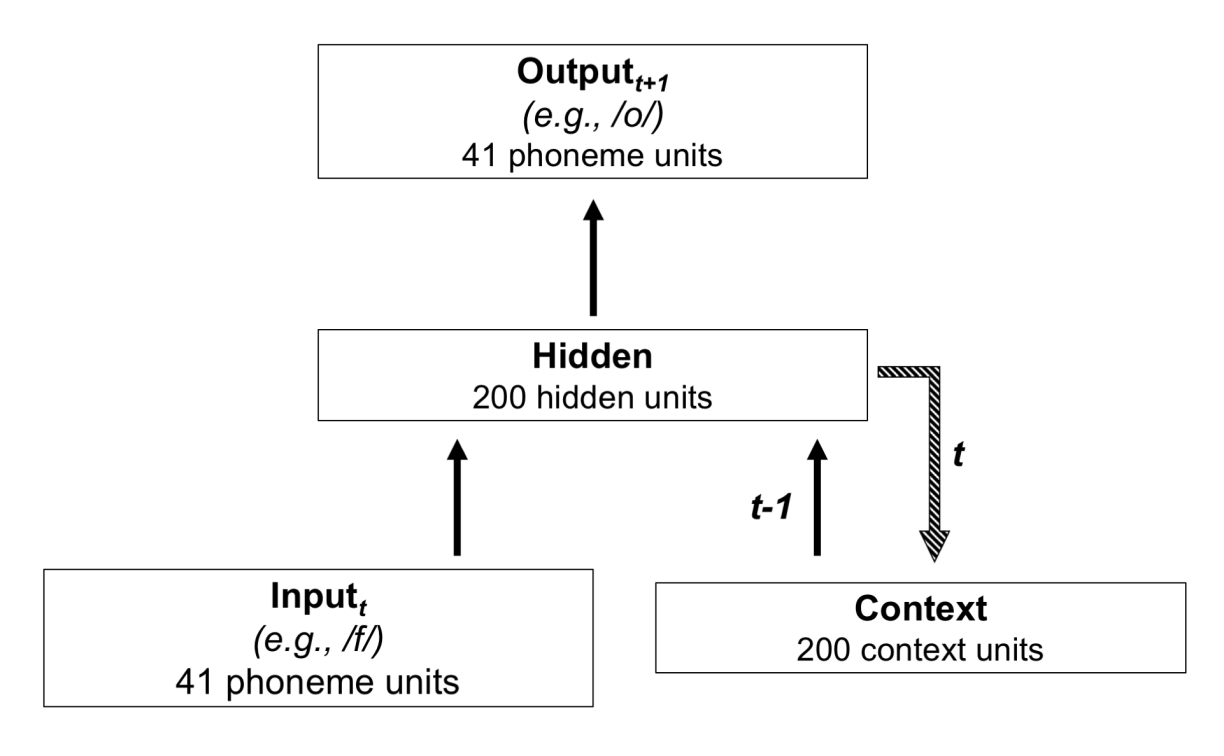


**Figure S1.** Architecture of an SRN built to predict the phoneme at time step *t+1* based on phonemic input at time *t*. In predicting the word *formula*, for instance, the model would predict the phoneme /o/ at time step *t+1* based on the input /f/ at time step *t*. The model maintains memory for previous hidden states by copying the states of the hidden units to a set of context nodes, which are fed back to the hidden layer at a one-cycle delay.

We first built an SRN that was designed to predict the next phoneme in a word based on the phonemes it had previously encountered (Figure S1). This SRN took phonemes as input (41 localist nodes, one for each phoneme) and had feedforward connections to a hidden layer (200 hidden units) and subsequently to an output phoneme layer (41 localist nodes). At each time step, the exact states of the hidden nodes were copied and fed to a set of context nodes, which in turn fed back to the hidden layer with a one-cycle delay. The context layer provides a form of memory that enables the SRN to become sensitive to contingencies that span multiple time steps.

The model’s initial lexicon was established using the same 37.6k words from the English Lexicon Project (Balota et al., 2007) as used in our implementation of the Gagnepain, Henson and Davis (2012) model. During each epoch, every word in the lexicon had the opportunity to be presented once; however, in order to capture differences in lexical frequency between items, the probability *p* of a word actually being presented on a particular epoch was defined as $p=0.05 \times ln(F)+0.1$, where *F* represents the number of occurrences in the Lund and Burgess (1996) corpus. We used backpropagation of error to establish the model’s weights: At each time step, the observed output pattern was compared to the desired activation (1.0 for the desired phoneme, 0.0 for all others). Backpropagation entails assignment of credit and blame to the different weighted connections in the network and involves calculating what changes in the model’s weights would allow the network to more closely approximate the desired activation pattern if the same input sequence were presented again. Approximately 10,000 epochs were used to establish the correct weights for the pre-training phase. We opted to use a relatively large number of epochs since there is not a single output pattern for a given input; rather, the model’s prediction of the upcoming phoneme is stochastic, based upon which phonemes have been presented in the past several steps. Notably, during each training epoch, the model was presented with a continuous set of phonemes (i.e., without indication of word boundaries), so the choice to use a large number of epochs was motivated by a desire for phoneme predictions to reflect the entire lexicon.

To simulate word learning, we trained the model for an additional 50 epochs on the 54 novel words; following the approach of Gagnepain et al. (2012), novel words were assigned the same frequency as their associated source word, and the probability of a novel word appearing in a given epoch was defined as described above. Note that as in the previous set of simulations, we counterbalanced which specific nonword (e.g., *formubo* or *formuty*) served as the novel word and report the average performance (canceling out potential differences due to such factors as the size and composition of lexical neighborhoods). A different random order of items was used during each epoch. The decision to train for an additional 50 epochs was motivated by pilot testing; with relatively few (e.g., 10) epochs of additional training, the model did not learn the novel words, but with too many (e.g., 100) epochs, performance on source words deteriorated.

Results from the SRN simulations are shown in Figure S2. From the top panel, it is clear that the SRN exhibits predictive processing, since the model can correctly predict upcoming phonemes (e.g., /l/ is well predicted after the model receives *formu*- as input). We note that the activation levels are substantially lower than might be predicted by the probabilities computed in the Gagnepain et al. (2012) model (see Figure 1 in the main text). We suspect that this is primarily because the SRN is presented with a continuous stream of phonemes, and the absence of word boundaries likely has an influence on phoneme probabilities; by contrast, the mathematical model used by Gagnepain et al. assumes that word segmentation has already occurred. The relatively low activation levels may also reflect imperfect lexical knowledge or limitations of short-term memory (as achieved through the context layer). We could better approximate probabilities if we were to transform the activations to response probabilities using the Luce choice rule (Luce, 1959), as is frequently done with the TRACE model (e.g., Allopenna, Magnuson & Tanenhaus, 1998; McClelland & Elman, 1986), but activations are arguably a more transparent index of model behavior. Nonetheless, it is striking that the model shows the expected relative patterns of activation for source, novel, and baseline items before and after training.

In the bottom panel, we show the prediction error (mean squared error) used by the model during training. As expected, we found that the SRN satisfied our three empirical targets. Specifically:

1. Prior to training on the novel words, the post-DP prediction error was high for both types of nonwords and low for the source words.
2. Following training, the post-DP phonemes in the novel words (e.g., -*bo* in *formubo*) were associated with decreased prediction error.
3. An influence of word learning was seen for pre-DP phonemes, which were associated with slightly higher error values prior to training (mean: 0.152) than after training (mean: 0.147).

In other words, the SRN shows reduced signal for expected inputs compared to unexpected ones, as measured in the error signal.


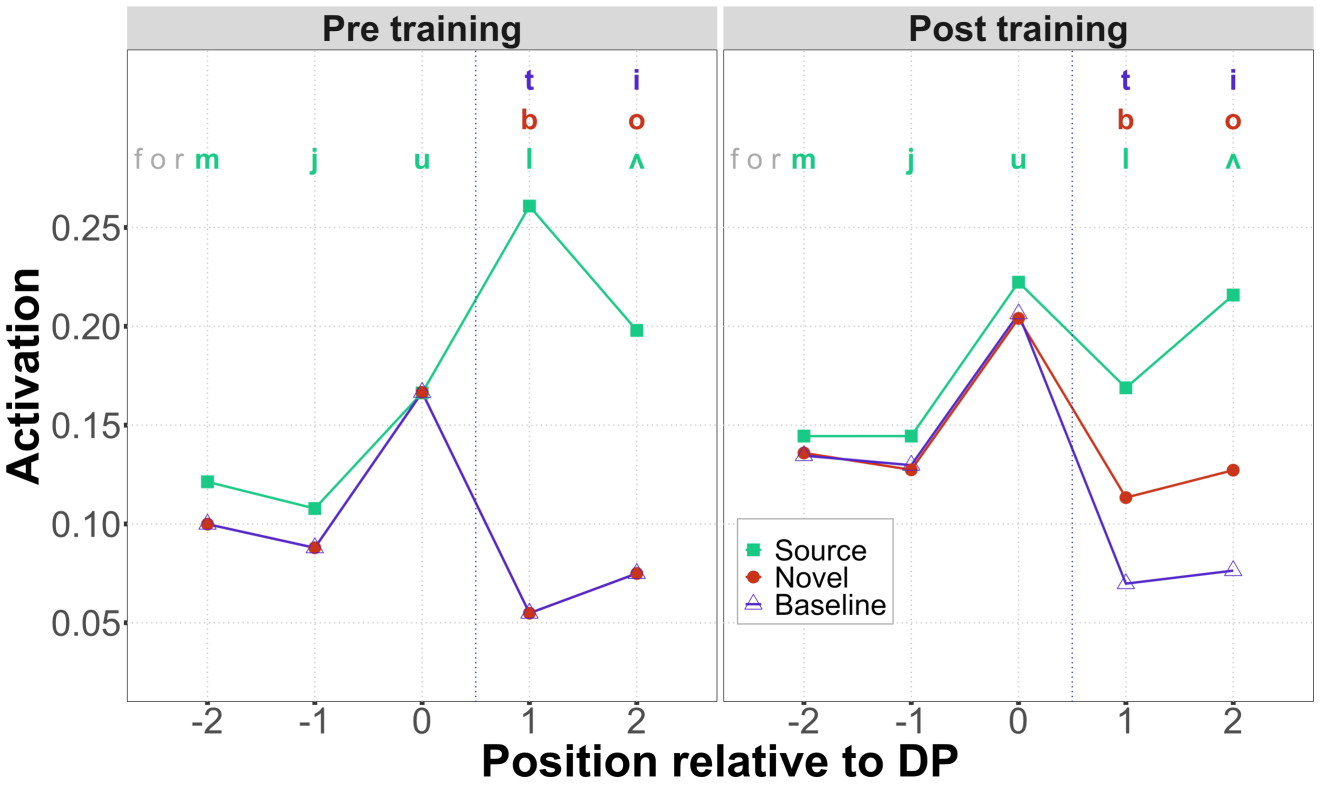


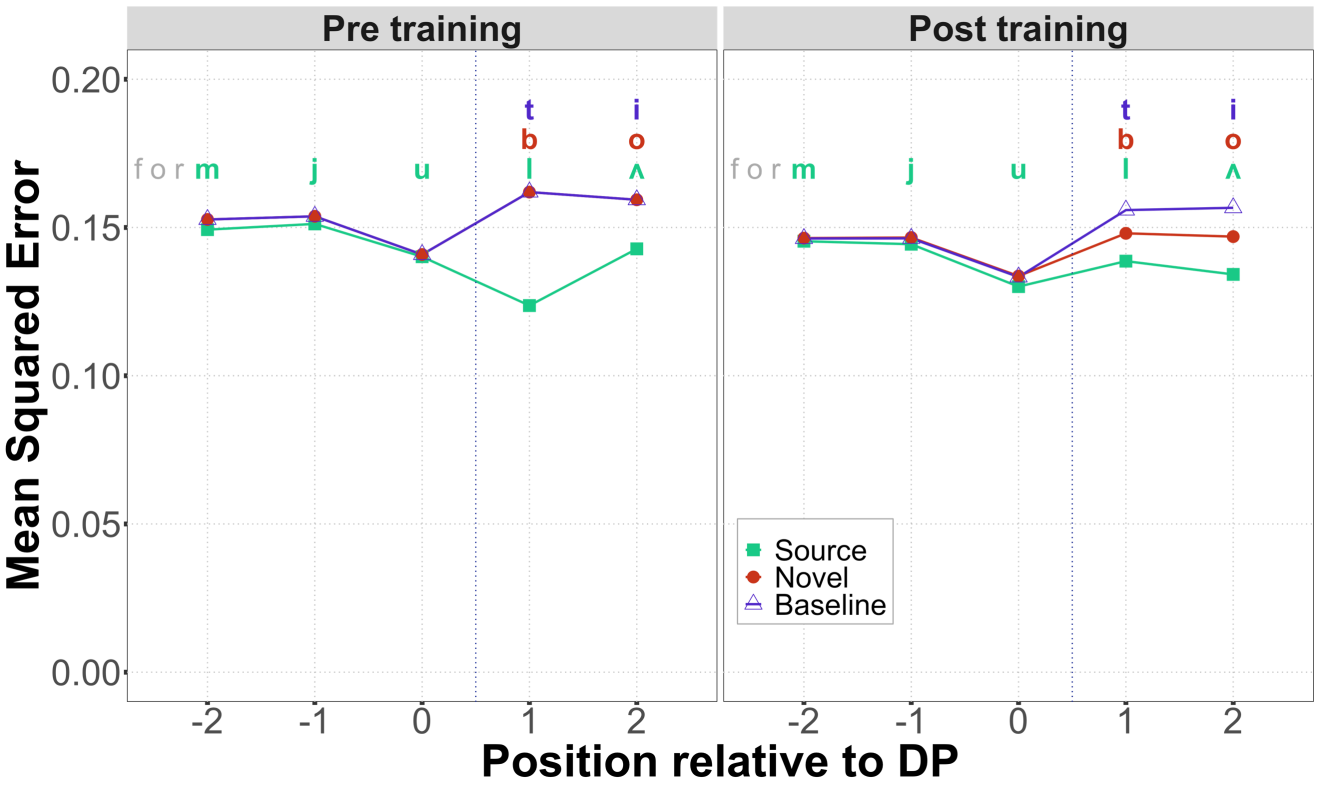


**Figure S2.** (Top) Phoneme-by-phoneme SRN output activations indicating how strongly the model predicted each upcoming phoneme. The model shows predictive processing insofar as activation levels directly reflect the probability of upcoming segments. (Bottom) Prediction error from the SRN, computed as mean squared error between desired and observed activation patterns. The model uses backpropagation of this error signal to train its weights.

***Additional TRACE Results***

In the novel word learning literature, a key behavioral finding is that when a novel word (e.g., *cathedruke*) is integrated into the lexicon, listeners endure increased lexical competition for the associated source word (e.g., *cathedral*) as measured in a pause-detection task (Gaskell & Dumay, 2003). Our TRACE simulations were consistent with these previous data, as we observed a delay in the recognition of the source word after training compared to before (Figure S3).


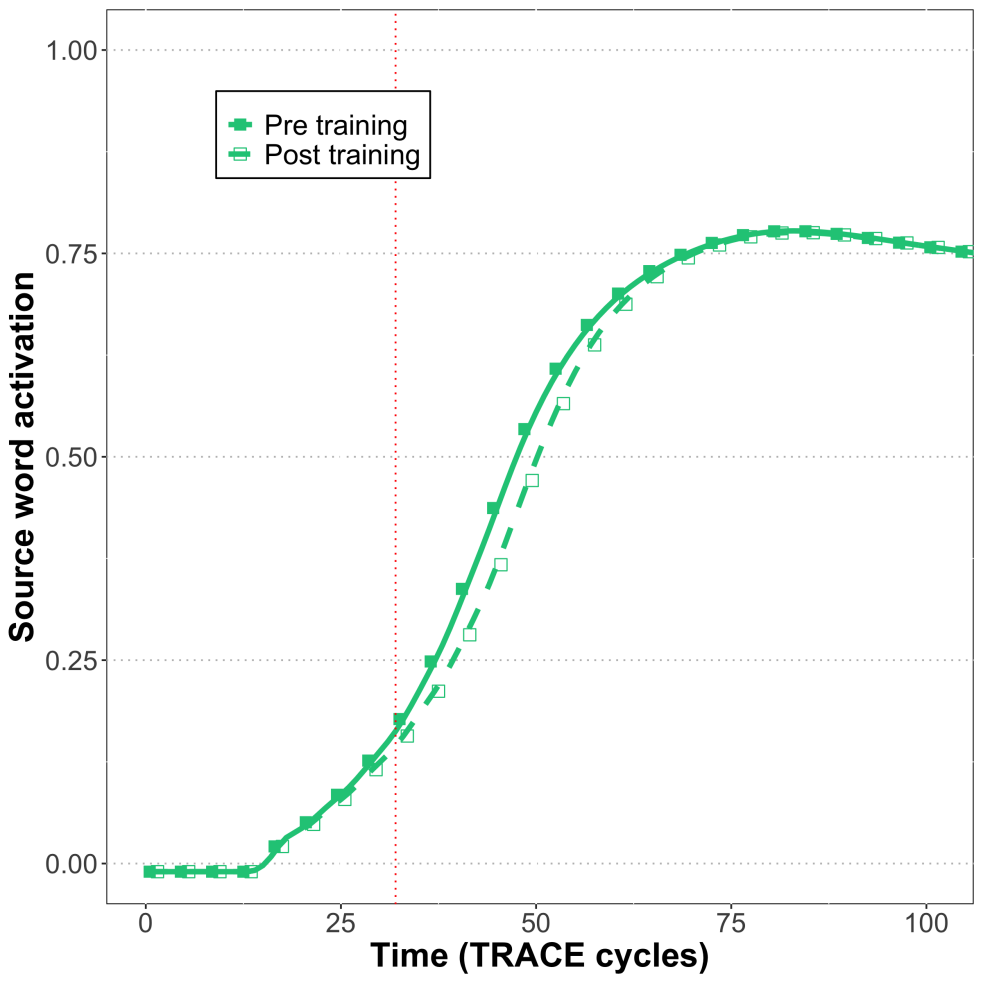


**Figure S3.** Time course for the activation of a source word (e.g., /partli/) in TRACE, both before (solid line with closed squares) and after (dashed line with open squares) the addition of a novel word (/partk^/) into the lexicon.

**Additional References**

Luce, R. D. (1959). *Individual Choice Behavior: A Theoretical Analysis*. New York: Wiley.

Lund, K., & Burgess, C. (1996). Producing high-dimensional semantic spaces from lexical co-occurrence. *Behavior Research Methods, Instruments, and Computers*, *28*(2), 203–208.
